# Magneto-Dielectric Effects in Polyurethane Sponge Modified with Carbonyl Iron for Applications in Low-Cost Magnetic Sensors

**DOI:** 10.3390/polym14102062

**Published:** 2022-05-18

**Authors:** Ioan Bica, Gabriela-Eugenia Iacobescu

**Affiliations:** 1 Advanced Environmental Research Institute, West University of Timisoara, Bd. V. Parvan, Nr. 4, 300223 Timisoara, Romania; ioan.bica@e-uvt.ro; 2 Department of Physics, University of Craiova, Str. A. I. Cuza, Nr. 13, 200585 Craiova, Romania

**Keywords:** polyurethane sponge, carbonyl iron, cylindrical capacitor, relative dielectric permittivity, dielectric loss coefficient, static magnetic field

## Abstract

In this study, magnetizable polyurethane sponges (MSs) were obtained from commercial absorbent polyurethane sponges (PSs) doped with carbonyl iron microparticles (CIPs). Based on MSs, we manufactured cylindrical capacitors (CCs). The CCs were subjected to both a magnetic field and an alternating electric field, with a frequency of f=1 kHz. Using an RLC bridge, we measured the series electric capacitance, $C_{s}$, and the tangent of the loss angle, $D_{s}$. From the functions $C_{s}=C_{s}{\left(\delta \right)}_{CCs}$ and $D_{s}=D_{s}{\left(\delta \right)}_{CCs}$, we extracted the components of the complex dielectric permittivity. It was found that the CIPs embedded in the MS matrix aggregated, leading to magneto-dielectric effects such as the enhancement of the complex dielectric permittivity components when applying the magnetic field as a principal effect and the enhancement of the electric capacitance and time constant of the capacitors as a secondary effect. The obtained results represent landmarks in the realization of low-cost magnetic field sensors, deformation and mechanical stress transducers in the robotics industry, etc.

## 1. Introduction

Large and recent scientific studies were dedicated to developing new tactile devices with high flexibility and sensitivity for the production of equipment that can detect mechanical deformations and stresses [1,2,3,4]. One of the main components of such a device is a smart material capable of measuring physical quantities such as force, displacement, heat, etc., before turning them into an electrical signal, which can then be used in an electrical circuit or microprocessor to generate a readable response for an actuator.

Among the few types of smart materials available to date is the porous polymeric matrix (PPM) containing magnetizable particles (MPs). PPM is a polyurethane sponge [1,5,6], and MP particles can be carbonyl iron microparticles [1,6], iron microparticles [7], magnetite nanoparticles [8,9], etc. The assembly of PPM with MPs forms what we call a magnetizable sponge (MS).

When subjected to an external magnetic field, the elastic properties of MS change reversibly [10,11,12]. When the magnetic field is applied the particles from PS transform in magnetic dipoles and arrange in chains along the magnetic field lines. During this process, the mechanical and rheological properties of MSs, as the components of the complex elasticity modulus, can be controlled by changing magnetic field intensities [13,14,15].

As reported in our previous study [16,17,18], magnetorheological suspensions (MRSs) based on silicone oil (SO) and CIPs can be well absorbed in PPM matrices. As a result, we obtained hybrid magnetorheological suspensions (hMRSs). When subjected to a magnetic field, the CIPs embedded in hMRSs form magnetic dipoles that align parallel with the applied magnetic field. During the orientation of magnetic dipoles, the physical characteristics of hMRSs [16,17,18] change drastically. This property of hMRSs is useful for manufacturing electrical devices [19], sensors and transducers for deformations and mechanical stresses [20], medical devices [21], etc.

On the other side, electromagnetic and magnetic fields are considered polluting factors [22,23]. In order to be notified of possible overruns, it is necessary to use materials for which their physical properties are sensitive to these fields. In our present study, we study the magneto-dielectric effects in MSs made from a commercially used polymeric sponge that is electrostatically doped with three different amounts of CIPs. Cylindrical capacitors (CCs) were manufactured from MSs. The equivalent series’ electrical capacity $C_{s}$ and the tangent of the dielectric loss angle $D_{s}$ of CCs were measured for three distinct values of the volume fraction $\Phi_{CI}$ of CIPs, and CCs were simultaneously subjected to a sinusoidal electric field, with a frequency of f=1 kHz and a magnetic field intensity gradient, δ. As a result of the formation of aggregates in the volume of MS sponges, functions $C_{s}=C_{s}{\left(\delta \right)}_{\Phi_{CI}}$ and $D_{s}=D_{s}{\left(\delta \right)}_{\Phi_{CI}}$ were found to be influenced by the values of the volume fractions $\Phi_{CI}$ and by the values of the magnetic field strength gradient δ. The experimental data indicate that the dispersion and absorption of electricity are controllable from the values of the sizes $\Phi_{CI}$ and δ.

The results obtained in this study could be useful in research activities exploring the realization of magnetic field sensors for protection against the magnetic fields of the control blocks of some technological processes and in the manufacture of sensors intended for the production of robots.

## 2. Materials and Methods

### 2.1. Manufacturing Magnetizable Polyurethane Sponges (MSs)

**Figure 1:**
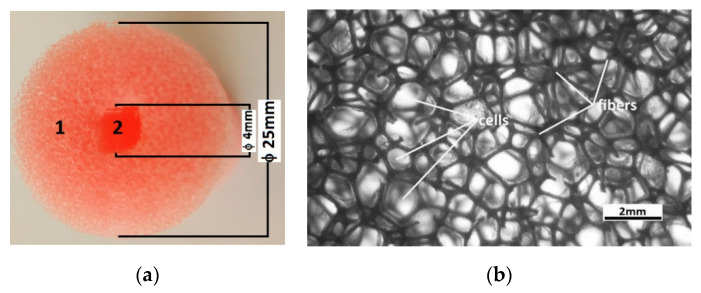
(**a**) Cross-section of the PS: (1) sponge body and (2) internal cylinder; (**b**) sponge body image captured with an XREC digital microscope.

**Figure 2:**
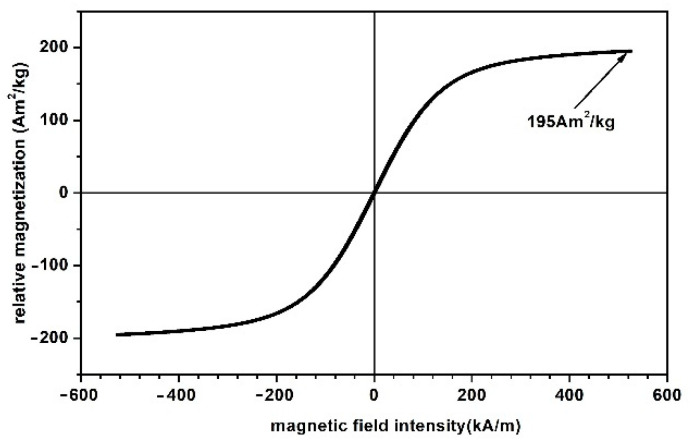
Magnetization slope for CIPs.

The materials used for the production of magnetizable sponges (MSs) were the following:

(a) The polymeric porous matrix (PPM) from polyurethane sponges (PSs): PSs were produced in China and distributed by SC Just Master Activities SRL, Bucharest, Romania. Each PS had the shape of a porous cylinder (Figure 1a). The shapes of the pores and fibers were visualized using a digital microscope (Figure 1b). The length of the PS was L=60 mm, the exterior diameter was D=25 mm, and the interior diameter was d=4 mm. Thus, the volume of the PPM matrix was $V_{PPM}=0.25\cdot \pi \cdot \left(D^{2}-d^{2}\right)\cdot L=28.6984{\;\mathrm{cm}}^{3}$. The mass of the PPM matrix was measured using an analytical balance type ALN60 produced by Axis, Gdańsk Poland, and found to be $m_{PPM}=0.6238\;g.$ Calculating the mass density of the PPM matrix at a room temperature of 24 °C, we obtained $\rho_{PPM}=m_{PPM}/V_{PPM}\approx 0.0217\;g/{\mathrm{cm}}^{3}$.
(b) The carbonyl iron microparticles (CIPs) were produced by Sigma-Aldrich (St. Louis, MO, USA) and sold in powdered form with a medium diameter of $\;d_{CIP}=5\;\mu m$. At a temperature of 24 °C, the CIP microparticle density was $\rho_{CIP}=7.86\;g/{\mathrm{cm}}^{3}$. Figure 2 shows the magnetization slope for CIPs obtained using the experimental setup in [24]. The maximum specific magnetization of CIPs was $195{\;\mathrm{Am}}^{2}/\mathrm{kg}$ for a magnetic field intensity of 520 kA/m.

The manufacture of MSs was performed in 6 stages.

Stage 1: From the PS, we cut three identical cylinders with a weight of $m_{PPM}=0.175\;g$ each. The cylinder mass was measured using an analytical balance type ALN60 produced by AXIS, Gdańsk, Poland.

Stage 2: We introduced the PSs into distilled water, and after 120 s, we transferred them into Petri dishes. In about 600 s, excess water was drained from the sponge. Using the above-mentioned analytical balance, we measured the water-soaked sponge weights and calculated the average value, which was found to be $m_{PPMw}=5.186\;g$. Then, the weight of the water soaked into PSs was calculated as $m_{w}=m_{PPMw}-m_{PPM}=5.011\;g\approx 5\;g$. The volume occupied by water in the PPM matrix was $V_{w}=m_{w}/\rho_{w}=5{\;\mathrm{cm}}^{3}$, and we considered the distilled water density at 24 °C as $\rho_{w}=1\;g/{\mathrm{cm}}^{3}$. We assumed that $V_{w}≅V_{a}$, where $V_{a}$ is the volume of the PPM matrix cells. Then, for the dried sponges, the weight of the air from the sponge cells was determined as $m_{a}=\rho_{a}\cdot V_{g}\approx 0.006\;g$, with air density at room temperature of $\rho_{a}=0.00120\;g/{\mathrm{cm}}^{3}$. The PPM matrix fiber weights, as shown in Figure 1, were estimated as $m_{PF}=m_{PPM}-m_{a}=0.169\;g$. The volume of the sponge in Figure 1 was calculated with Equation $V_{PS}=\left(\pi /4\right)\cdot \left(D^{2}-d^{2}\right)\cdot L$. Using D=2.5 cm, d=0.4 cm and L=1.8 cm, we obtained $V_{PS}=8.6096\;{\mathrm{cm}}^{3}$. The total fiber volume in the PS was calculated as $V_{PF}=V_{PS}-V_{a}=3.6096{\;\mathrm{cm}}^{3}$.

**Figure 3:**
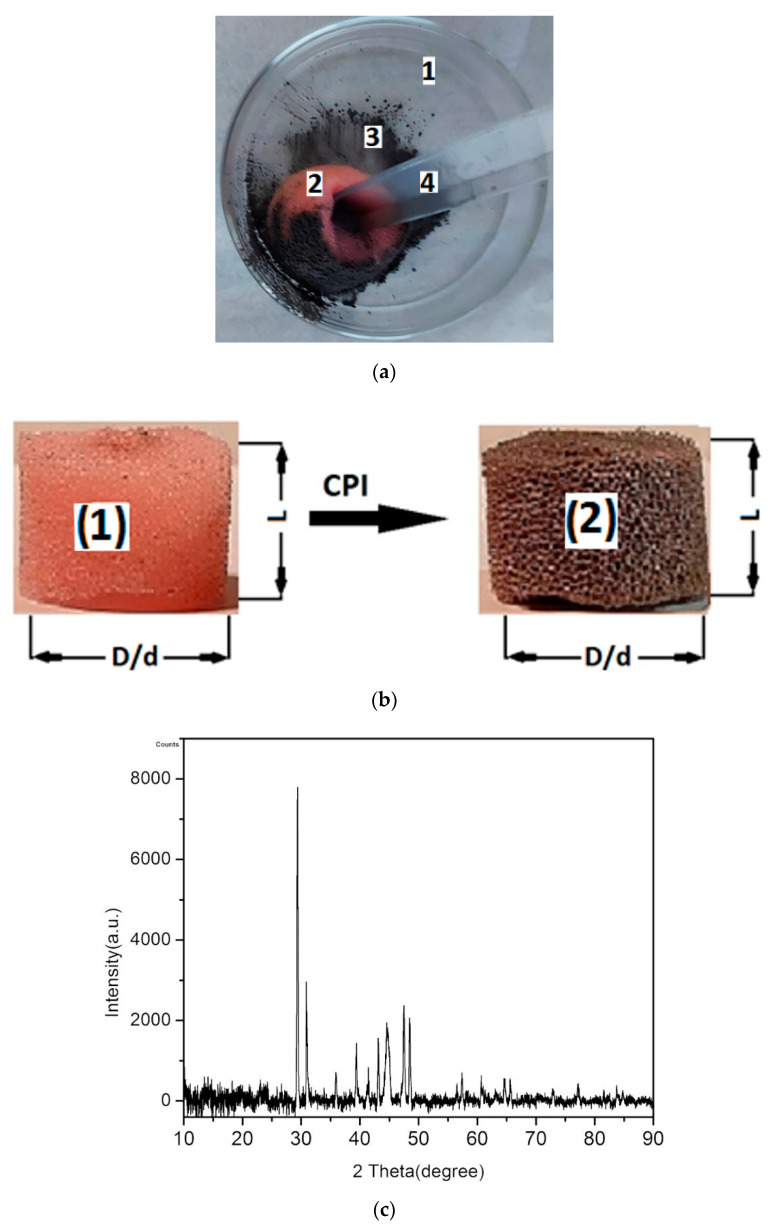
(**a**) MS sponge manufacturing (1—Petri dish; 2—PS; 3—CIPs; 4—glass rod); (**b**) PS before filling (1) and after filling (2) with CIPs; (**c**) XRD analysis of MS.

Stage 3: We introduced the PS with a weight of $m_{PPM}=0.175\;g$ and the CIPs in a Petri dish, as shown in Figure 3a. Using a glass rod, we performed repeated deformations of the PS, which determined the absorption of CIPs in the PPM matrix. This procedure was repeated since no CIPs flowed from the sponge when shaking, thereby obtaining a magnetizable sponge and MSs with sizes of D=2.5 cm, d=0.4 cm, and L=1.8 cm, as shown in Figure 3b. In the same manner, we produced three sponge samples uniformly doped with CIPs. The crystallographic structure of MSs is shown in Figure 3c. The crystal phase of the MSs was investigated by a PANalytical diffractometer, with Cu-K_α radiation (λ=0.15406 mm) and 2θ range from 10° to 90°. We can obsesrve from Figure 3c that MSs have a CIPs specific crystalline phase [25,26] and an amorphous phase specific to polyurethane nanofibers [27]. CIPs are neither thermally nor chemically treated based on the studies performed in [28]. The peaks in Figure 3c represent nanometric crystallites of the α-Fe type.

Using the analytical balance, we measured the weights of the sponges uniformly doped with CIPs. Considering $m_{PF}=0.169\;g$, we then calculated $m_{CIP}$ for the three samples. Table 1 shows the weights, volumes and volumetric fractions for the samples $MS_{i}$, where i=1, 2, 3 is the sample number. The volumes occupied by the CIPs were calculated using $V_{CIP}=m_{CIP}/\rho_{CIP}$. The air volume was calculated as $V_{a}=V_{PS}-\left(V_{PF}+V_{CIP}\right)$. The volumetric fractions $\Phi_{PF},$ $\Phi_{CIP}$ and $\Phi_{a}$ were calculated using a reference of $V_{PS}=8.6096{\;\mathrm{cm}}^{3}$.

### 2.2. Manufacturing Cylindrical Capacitors (CCs)

The materials used for cylindrical capacitor manufacturing were the following:

(a) A copper sheet (FCu) with a size of 500 × 500 × 0.50 mm (composition code CW004A (CuETP), product code HOBC005, and delivered by emag.ro);
(b) $MS_{i}$ samples, described in Table 1.

**Figure 4:**
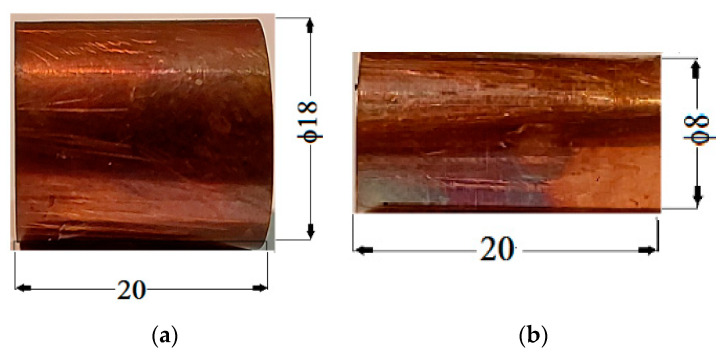
The copper armatures of the DC capacitors: (**a**) outer cylinder; (**b**) inner cylinder.

For manufacture CC armatures, from one FCu foil, we cut three pieces with dimensions of 63 × 20 × 0.50 mm for the external cylinders (Figure 4a) and three pieces with dimensions of 20 × 20 × 0.50 mm for the external cylinders (Figure 4b).

**Figure 5:**
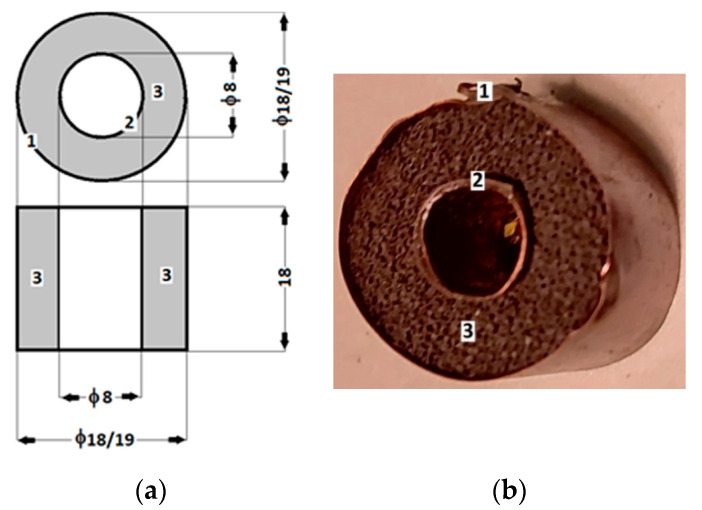
Manufacture of the cylindrical capacitors: (**a**) sketch of the shape and dimensions; (**b**) photographic image of the capacitor (1—external cylinder; 2—internal cylinder; 3—MS sponge).

Each MS sample was forcibly inserted between the copper armatures, achieving the overall configuration shown in Figure 5a. At the end of this process, the CCs from Figure 5b were obtained.

From each magnetizable sponge, $MS_{i}$, we obtained a corresponding $CC_{i}$, i=1, 2, 3 capacitor.

### 2.3. Experimental Setup

**Figure 6:**
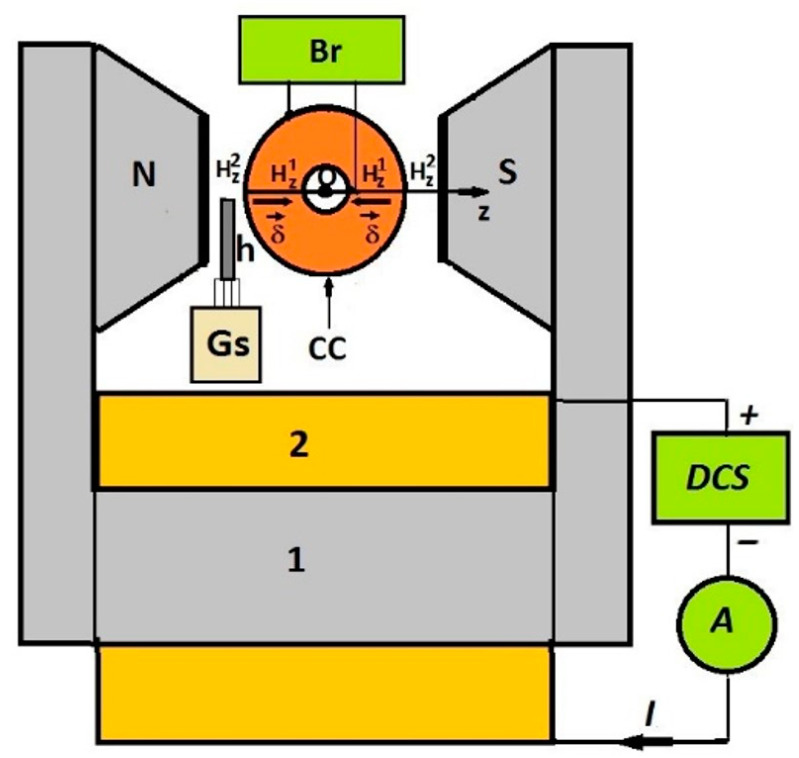
Experimental setup (overall configuration) consisting of an electromagnet (with the following components: 1—magnetic yoke; 2—coil; N and S—magnetic poles); CC—cylindrical capacitor; Br—RLC bridge; Gs—Gaussmeter; h—Hall probe; DCS—direct current source; A—direct current ammeter; I—electric current intensity; Oz—coordinate axis; $H_{z}^{1}$ and $H_{z}^{2}$—magnetic field intensities; $\vec{\delta }$—vector of the magnetic field intensity gradient.

Figure 6 shows the overall experimental setup used to study the magnetodielectric effects induced in MSs by a magnetic field superimposed over an electric field with a medium frequency.

**Figure 7:**
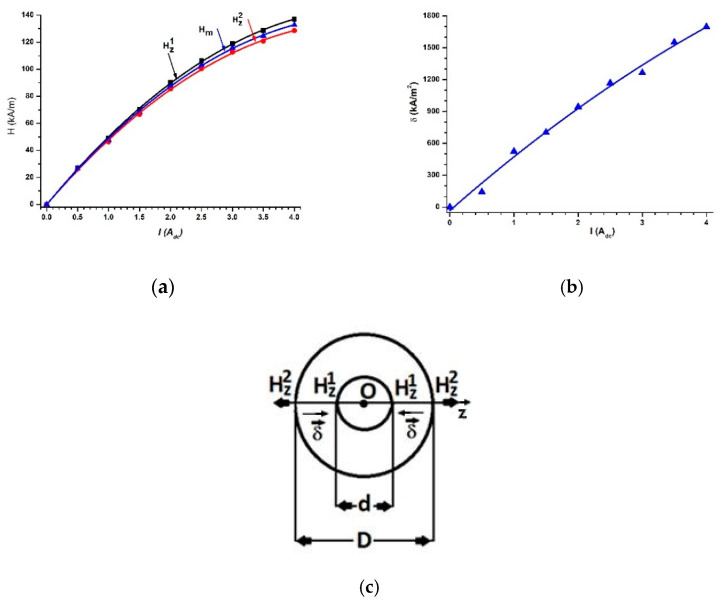
(**a**) The magnetic field intensities $H_{z}^{1}$, $H_{z}^{2}$ and $H_{m}$ as functions of the electric current intensity, I; (**b**) dependence of the magnetic field intensity gradient, δ, on the electric current intensity, I; (**c**) cross-section of CCs, where *D* is the external diameter and *d* is the internal diameter.

The experimental setup included an electromagnet powered by a current source and a DCS (type RXN-3020D, from Electronics Co., Ltd., Guangdong, China) in series with an ammeter, A, as part of a Mastech digital multimeter (type MY64, from Shenzhen New Huayi Instrument Co., Ltd., Shenzhen, China). By adjusting the electric current intensity, I, through the electromagnet coil, we controlled values $H_{z}^{1}$ and $H_{z}^{2}$ of the magnetic field intensities between the N and S poles of the electromagnet along the Oz axis and, consequently, the average values of the magnetic field intensity between the armatures of the capacitor, CC (i.e., $H_{m}=0.5\left(H_{z}^{1}+H_{z}^{2}\right)$). The values of the magnetic field intensity were measured with a Gauss-meter, Gs (type DX-102, from DexingMagnet, Xiamen, China). The capacitor, CC, fixed between magnetic poles N and S, was connected to the RLC bridge, Br (type CHY-41R, from FIREMATE, Taiwan, China). The non-uniformity of the magnetic field generated a gradient of the magnetic field intensity between the capacitor armatures, CC, calculated with the equation $\delta =2\;\left(H_{z}^{2}-H_{z}^{1}\right)/\left(D-d\right)$. Using *D* = 18 mm, *d* = 8 mm, and the values $H_{z}^{1}=H_{z}^{1}\left(I\right)$ and $H_{z}^{2}=H_{z}^{2}\left(I\right)$ from Figure 7a, we plotted the dependence δ=δ(I), as shown in Figure 7b.

## 3. Results and Discussion

### 3.1. Measurements and Experimental Data

The capacitors CCs were inserted, one by one, between the N and S poles of the electromagnet and connected to the bridge, Br (Figure 6). The bridge, Br, was fixed at an operating frequency of f=1 kHz.

By varying the intensity of the electric current, I, the values of the magnetic field intensity gradient, δ, became fixed (after each change in the electric current intensity, and a waiting period of 120 s was employed to ensure cessation of the transient regime, and then the data were recorded). Using the CHY-41R bridge, the series equivalent electrical capacity, $C_{s}$, and the tangent of the dielectric loss angle, $D_{s}$, of the CCs were measured.

**Figure 8:**
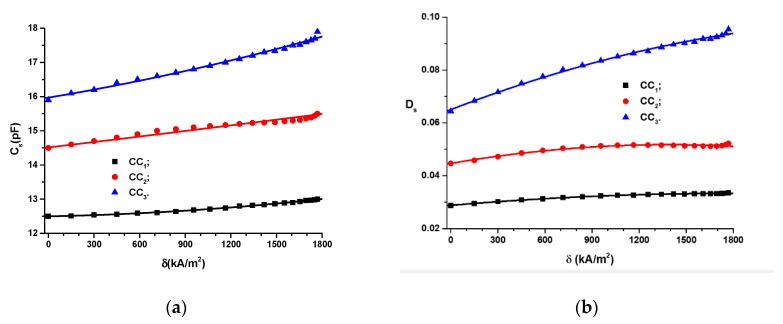
(**a**) The electric capacitance, $C_{s}$, vs. the magnetic field intensity gradient, *δ*, for different volumetric fractions; (**b**) the tangent of the dielectric loss, $D_{s}$, vs. the magnetic field intensity gradient, *δ*, for different volumetric fractions (dots are experimental data, and lines are the second-order polynomial fit).

The measured values are graphically represented in Figure 8. In Figure 8, we can observe that, in the absence of the magnetic field, the magnitudes of the equivalent series electric capacitance, $C_{s}$, and the tangent of the dielectric loss, $D_{s}$, increased with an increase in the volumetric fractions, $\Phi_{CIP}$.

For fixed values of the volumetric fractions, $\Phi_{CIP}$, the equivalent series electric capacitance, $C_{s}$, depends on *δ* as follows:

(1) $$C_{s}=C_{s_{0}}+\alpha_{C_{s}}\delta +\beta_{C_{s}}\delta^{2}$$

where $C_{s_{0}},\;\alpha_{C_{s}}$ and $\beta_{C_{s}}$ parameters resulting from the second-order fitting of data from Figure 8a and are summarized in Table 2. δ is the modulus of the magnetic field intensity gradient.

For fixed values of the volumetric fractions, $\Phi_{CIP}$, the tangent of the dielectric loss, $D_{s}$, depends on *δ* as follows:

(2) $$D_{s}=D_{s_{0}}+\alpha_{D}\delta -\beta_{D_{s}}\cdot \delta^{2}$$

where $D_{s_{0}}$, $\alpha_{D_{s}}$ and $\beta_{D_{s}}$ result from the second-order fitting of data from Figure 8b and are summarized in Table 3. δ is the modulus of the magnetic field intensity gradient.

Figure 8 and Table 2 and Table 3 show that the second-order polynomial functions accurately approximated the experimental data. Figure 8a shows that CCs can be considered as magnetic field sensors.

### 3.2. Comparison with Data Reported in the Literature

The comparison of the experimental data from Figure 8a with those reported in the literature is made based on the following considerations.

(a) Electrical devices (EDs) are manufactured using natural and artificial fiber matrices with CIPs and additives (silicone oil, barium titanate nanoparticles and iron oxide microfibers).

(b) Measurements of equivalent electrical capacities of EDs are performed in an alternating electric field of frequency *f* = 1 kHz, superimposed over the static magnetic field.

Based on these criteria, Table 4 presents comparative values for equivalent electrical capacities of EDs.

As we can see from Table 4, the equivalent series ($C_{s}$) and parallel ($C_{p\;}$) equivalent electrical scheme of the EDs are determined by the value of the electrical capacity. From the same table, we observe that the values of the equivalent electrical capacity increase with the increase in the amount of CIPs. For the same amount of CIPs, the equivalent electrical capacity values of the EDs are significantly influenced by the H values of the magnetic field strength and the additives used (nTB and *μF*), except for those in the MSs (Figure 8a).

### 3.3. Equivalent Electrical Scheme of CCs

**Figure 9:**
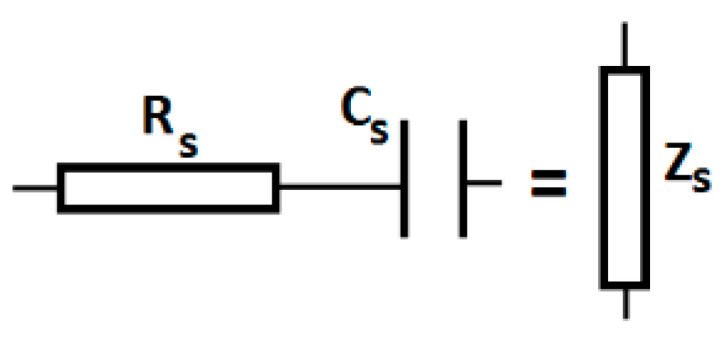
Equivalent electrical circuit for the real CCs: $R_{s}$, resistor; $C_{s}$, capacitance; $Z_{s}$, series impedance.

The results plotted in Figure 8 suggest that CCs are real capacitors, for which its equivalent electrical circuit is represented in Figure 9.

In AC, the real electrical CCs have the following impedance:

(3) $$Z_{s}=\sqrt[{2}]{R_{s}^{2}+X_{s}^{2}}$$

where $R_{s}$ and $X_{s}$ are the equivalent electrical resistance and reactive capacitance, respectively.

The magnitude of $X_{s}$ is calculated by the following equation:

(4) $$X_{s}=\frac{1}{2\pi fC_{s}}$$

where f is the AC field frequency, and $C_{s}$ is the equivalent electrical capacitance of the CCs.

**Figure 10:**
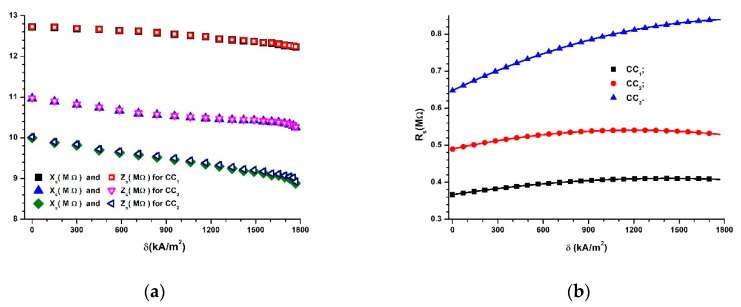
(**a**) Reactive capacitance, $X_{s}$, and impedance, $Z_{s}$, of CCs as functions of the magnetic field intensity gradient, δ; (**b**) the equivalent resistance, $R_{s}$, as a function of the magnetic field intensity gradient, δ.

By introducing f=1 kHz and the values $C_{s}=C_{s}{\left(\delta \right)}_{C_{i}},\;\left(i=1,\;2,\;3\right)$ from Figure 8a into Equation (4), we can obtain the dependences $X_{s}=X_{s}{\left(\delta \right)}_{C_{i}},\;\left(i=1,\;2,\;3\right)$ represented in Figure 10a.

The values of $C_{s}$, $D_{s}$ and $R_{s}$ are related by the following equation [32]:

(5) $$R_{s}=\frac{D_{s}}{2\pi fC_{s}}$$

where the notations are as outlined above.

Considering f=1 kHz, Equation (5) becomes the following.

(6) $$R_{s}\left(M\Omega \right)=\frac{159\cdot D_{s}}{C_{s}\left(\mathrm{pF}\right)}.$$

If we introduce the values of $C_{s}=C_{s}{\left(\delta \right)}_{C_{i}}$ from Figure 8a into Equation (6), along with the values of $D_{s}=D_{s}{\left(\delta \right)}_{C_{i}}$ from Figure 8b, and we can obtain the dependences $R_{s}=R_{s}{\left(\delta \right)}_{C_{i}},\;\left(i=1,\;2,\;3\right)$, as shown in Figure 10b.

Figure 10b shows that the electrical resistance increases with an increase in $\Phi_{CIP}$ in the absence of a magnetic field. However, when $\Phi_{CIP}$ values are fixed, $R_{s}$ increases when increasing the values of the magnetic field intensity gradient, δ.

If we introduce the values of $X_{s}$ from Figure 10a and the values of $R_{s}$ from Figure 10b into Equation (3), we can obtain the dependences $Z_{s}=Z_{s}{\left(\delta \right)}_{C_{i}},\;\left(i=1,\;2,\;3\right)$, as shown in Figure 10a.

It can be seen from Figure 10a that the values of $X_{s}$ and $Z_{s}$ decrease with an increase in $\Phi_{CIP}$ and δ.

Moreover, Figure 10a shows that, for i=1, 2, 3, the functions $X_{s}=X_{s}{\left(\delta \right)}_{C_{i}}$ and $Z_{s}=Z_{s}{\left(\delta \right)}_{C_{i}}$ overlap. This result suggests that, from an electrical point of view, the CC devices have predominant resistive characteristics.

Figure 10a, together with Figure 8a, proves that CCs have, from an electrical point of view, a resistive character, and they can be considered as magnetic field sensors.

Recent work [20] reported the manufacturing Eds with membranes based on cotton fabric with CIPs and barium titanate nanoparticles. The electrical response of the Eds, i.e., equivalent electrical resistance, equivalent electrical capacity and electrical voltage, measured at the Eds terminals, changes significantly with increasing mechanical compression. For the same mechanical compression, the sensitivity of the electrical response of EDs depends on whether it can be controlled by the magnetic field.

### 3.4. The Components of the Complex Dielectric Permittivity of MSs in the Absence of the Magnetic Field

In the absence of a magnetic field, the electrical capacity of the CCs is calculated using the following equation:

(7) $$C_{s_{0}}=\frac{2\pi \varepsilon_{0}\varepsilon^{\prime }L}{ln\left(\frac{R_{2}}{R_{1}}\right)}$$

where $\varepsilon_{0}$ is the vacuum dielectric constant, $\varepsilon^{\prime }$ is the relative dielectric permittivity, and $R_{2}=0.5\;D,\;R_{1}=0.5\;d$ and L are the radii and length of the MSs inside the CCs.

If we introduce $\varepsilon_{0}=8.854\mathrm{pF}/m$, $L=18\times 10^{-3}\;m$, $R_{2}=9\times 10^{-3}\;m$, and $R_{1}=4\times 10^{-3}\;m$ into Equation (7), we obtain the following.

(8) $$\varepsilon^{\prime }\approx 0.810\cdot C_{s_{0}}\left(pF\right).$$

If we introduce the values of $C_{s_{0}}$ from Table 2 into Equation (8), we obtain the values for $\varepsilon^{\prime }$ reported in Table 5.

As shown in Table 5, in the absence of a magnetic field, the quantities $\varepsilon^{\prime }$ are influenced by the volume fractions of the CIPs, of the microfibers, and, consequently, of the holes in the PS volume. Thus, with an increase in $\Phi_{CIP}$ and a decrease in $\Phi_{PF}$ and $\Phi_{a}$, the values of the relative dielectric permittivity, $\varepsilon^{\prime }$, increase (see Table 1).

Between the dielectric loss coefficient, $\varepsilon^{\prime \prime }$, the relative dielectric permittivity $\varepsilon^{\prime }$, and the tangent of the dielectric loss angle $D_{s}$, we can obtain the following equation [32].

(9) $$\varepsilon^{\prime \prime }=D_{s}\varepsilon^{\prime },\;\mathrm{for}\;t=0$$

In Equation (9), we introduce the values $D_{s}$ from Table 3 and the values $\varepsilon^{\prime }$ from Table 5 for each MS. In this manner, we obtain the values of $\varepsilon^{\prime \prime }$ for each corresponding sponge (Table 5).

It can be seen from Table 5 that an increase in the value of the volume fraction of CIPs in MSs, $\Phi_{CIP}$, increases the relative dielectric permittivity, $\varepsilon^{\prime }$, and thus increases the dielectric loss coefficient, $\varepsilon^{\prime \prime }$.

### 3.5. The Components of the Complex Dielectric Permittivity of MSs in the Presence of the Magnetic Field (Theoretical Model)

When an external magnetic field is applied, the CIPs transform into magnetic dipoles. The magnetic moment of a dipole $\vec{m}$, projected in the direction of the Oz axis and identical to that of $\vec{\delta }$, is calculated as follows [16,17,18,19,20,21]:

(10) $$m=0.5\pi d_{CIP}^{3}H_{m}$$

where $d_{CIP}$ is the average diameter of CIPs, and $H_{m}$ is the average value of the magnetic field intensity.

**Figure 11:**
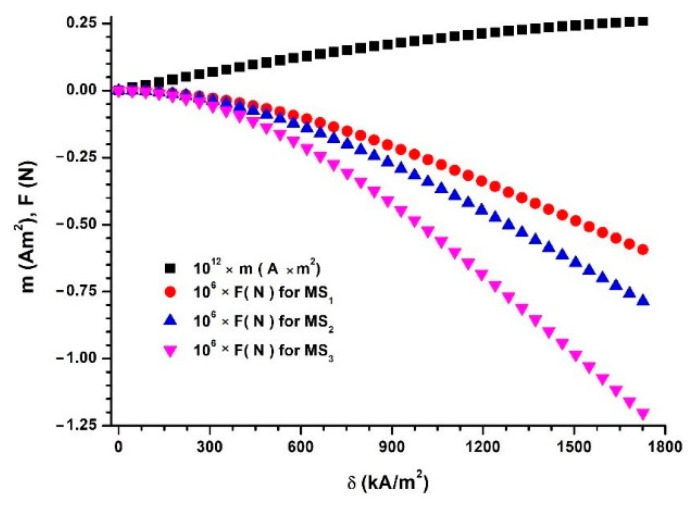
Magnetic moment, m, and magnetic force, $F_{m}$, between two identical and adjacent magnetic dipoles in MSs as functions of the magnetic field intensity gradient, δ.

If we introduce the values $d_{CIP}=5\;\mu m$ and $H_{m}=H_{m}\left(I\right)$ from Figure 7a into Equation (10), we obtain the dependence m=m(δ), as depicted in Figure 11.

As shown in Figure 11, the m values of the magnetic moment increase significantly with an increase in the magnetic field intensity gradient, δ.

Between two neighboring and identical dipoles, $\vec{m}$, magnetic interactions occur. The intensity of these interactions in the direction of the Oz axis can be calculated with the following relation [20,21]:

(11) $$F_{mz}=-\mu_{s}\mu_{0}m\delta$$

where $\mu_{s}\approx 1$ is the magnetic permeability of the PS, $\mu_{0}$ is the magnetic constant of the vacuum, m is the modulus of the magnetic moment vector and δ is the modulus of the vector of the magnetic field intensity gradient.

The number of dipoles in the volume of MSs can be approximated by the following equation:

(12) $$n=\frac{\Phi_{CIP}V_{MSs}}{V_{CIP}}=\frac{1.5\Phi_{CIP}\left(D^{2}-d^{2}\right)}{d_{CIP}^{3}}$$

where $\Phi_{CIP}$ is the volumetric fraction of dipoles $\vec{m}$; $V_{MSs}$ is the MS volume between the CC armatures; $V_{CIP}$ is the volume of one dipole $\vec{m}$; D and d are the internal and external diameters, respectively; L is the common length of two adjacent dipoles.

The number of dipoles in a chain in the direction of the Oz axis is calculated as follows:

(13) $$n_{1}=\frac{0.5\left(D-d\right)}{d_{CIP}}$$

where the notations are defined as shown above.

The number of magnetic dipole chains along the Oz axis can be defined by the equation $n_{2}=n/n_{1}$. Then, using Equations (12) and (13), we obtain the following.

(14) $$n_{2}=\frac{3\Phi_{CIP}\left(D+d\right)L}{d_{CIP}^{2}}$$

If the magnetic force in each magnetic dipole chain is $F_{mz}$, as given by Equation (11), then the magnetic force in the volume MSs is calculated as follows.

(15) $$F_{m}=n_{2}F_{mz}=3\mu_{s}\mu_{0}m\delta \;\frac{\Phi_{CIP}\left(D+d\right)L}{d_{CIP}^{2}}$$

Then, using $\mu_{s}=1$, $\mu_{0}=4\pi \times 10^{-7\;}H/m$, $\Phi_{CIP}$ from Table 1, D = $18\times 10^{-3}$ m, $d=8\times 10^{-3}$ m, $L=18\times 10^{-3}$ m, and $d_{CIP}=5\;\mu m,$ we can obtain the dependence $F_{m}=F_{m}\left(\delta \right)$ shown in Figure 11.

We can observe from Figure 11 that $F_{m}$ increases with an increase in $\Phi_{CIP}$. For fixed $\Phi_{CIP}$ values, the sizes $F_{m}$ decrease significantly with an increase in δ.

**Figure 12:**
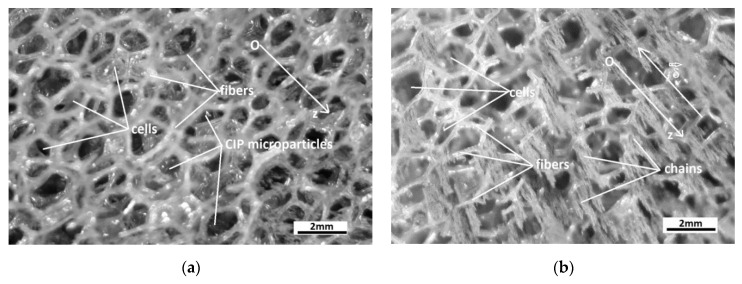
(**a**) MS sponges in the absence of a magnetic field; (**b**) MS when subjected to a magnetic field. The Oz axis has its origin on the central axis of the CCs, and $\vec{\delta }$ is the vector of magnetic field intensity gradient.

**Figure 13:**
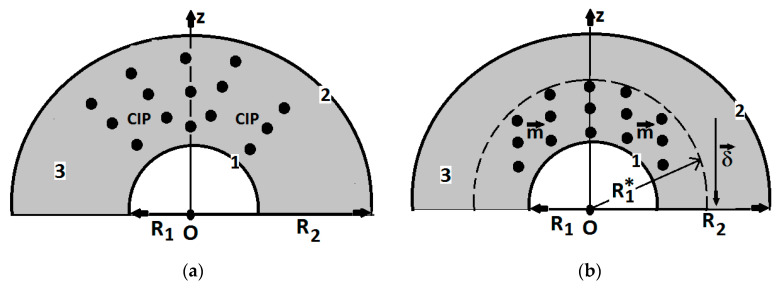
Solid microparticles in the polyurethane sponge matrix placed between the CC armatures (model): (**a**) in the moment of applying the magnetic field (t=0); (**b**) a while after the magnetic field was applied (t>0); 1 and 2—metallic armatures; 3—polyurethane sponge; CIP—carbonyl iron microparticles; $\vec{m}$—magnetic dipole; $\vec{\delta }$—the vector of magnetic field intensity gradient; Oz—coordinate axis; $R_{1}$—internal cylinder radius; $R_{2}$—external cylinder radius; $R_{1}^{*}$—the apparent radius.

We consider CIPs to be uniformly dispersed in the MS cells (Figure 12a and Figure 13a). When the magnetic field is applied in the direction of the Oz axis, CIPs transform into magnetic diploes, $\vec{m}$, and, after a certain time, the magnetic dipoles align in columns and concentrate in the direction of $\vec{\delta }$ (Figure 12b).

Under the action of $F_{m_{z}}$ much higher than electrostatic force, the dipole columns agglomerate in the immediate vicinity of the surface of the inner cylinder of the capacitor armature CCs (Figure 13b). The resulting effect is the increasing in radius from $R_{1}$ to a value $R_{1}^{*}$, which is called the apparent radius. When the magnetic field is canceled, under the action of the electrostatic force, CIPs will be attracted on the surface of the polyurethane fibers, and radius $R_{1}^{*}$ returns to the value $R_{1}$.

The formula for calculating the electrical capacity of CCs in the presence of a magnetic field is as follows:

(16) $$C_{s}=\frac{2\pi \varepsilon_{0}\varepsilon^{\prime }L}{ln\left(\frac{R_{2}}{R_{1}^{*}}\right)},\;\mathrm{at}\;a\;\mathrm{moment}\;t>0$$

where the notations are defined above.

From Equations (7) and (16), we can calculate $R_{1}^{*}$ as follows.

(17) $$R_{1}^{*}=R_{1}exp\left[2\pi \varepsilon_{0}\varepsilon^{\prime }\left(\frac{1}{C_{s_{0}}}-\frac{1}{C_{s}}\right)\right]$$

**Figure 14:**
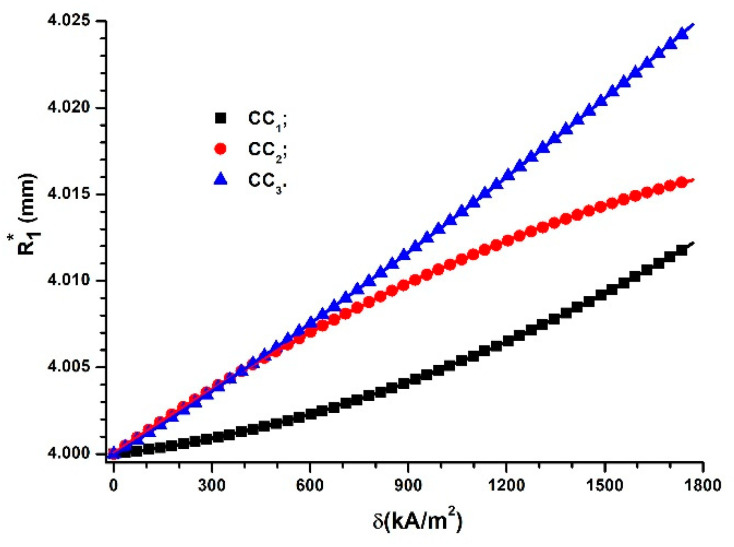
Apparent radius, $R_{1}^{*}$, of CCs as function of the gradient of magnetic field intensity, δ.

Then, with $R_{1}=4\;\mathrm{mm},$ $\varepsilon_{0}=8.845\mathrm{pF}/m\;$, $L=18\times 10^{-3}$ m, $\varepsilon^{\prime }$ from Table 3, and the functions $C_{s}=C_{s}{\left(\delta \right)}_{CC_{i}}$ (*i* = 1, 2 and 3) from Figure 8a (introduced in Equation (17)), we obtain the dependences $\;R_{1}^{*}=R_{1}^{*}{\left(\delta \right)}_{CC_{i}}$, where *i* = 1, 2 and 3 (Figure 14).

As we can observe from Figure 14, $R_{1}^{*}$ is dependent on the volumetric fraction of CIPs, $\Phi_{CIP}$. For values of $\delta \geq 750\mathrm{kA}/m^{2}$, the values of the apparent radius are distinct and increase with an increase in the magnetic field intensity gradient, δ. The metallic magnetizable phase is not uniformly distributed around the internal cylinder surface. Although the magnetic force, $F_{m}$, increases with the amount of the magnetizable phase, the presence of polymeric fibers retains the magnetizable CIPs.

The values of the electrical capacity, $C_{s}$, in Figure 8a correspond to an apparent relative dielectric permittivity between the armatures of the CCs, which we denote by $\varepsilon^{*}{}^{\prime }$. Thus, we can consider MSs to be materials characterized by apparent relative dielectric permittivity. Size $\varepsilon^{*}{}^{\prime }$ appears in dielectrics whenever an external interaction intervenes (i.e., static electric field, magnetic field, field of stresses and mechanical deformations and/or combinations thereof). When these causes are removed, the apparent relative dielectric permissibility returns to the value caused by the composition of the dielectric between the armatures of the DC capacitors, $\varepsilon^{\prime }$.

Based on the abovementioned factors, for times t>0, from the application of the magnetic field, the calculation formula for the electrical capacity of the CCs changes and becomes the following.

(18) $$C_{s}=\frac{2\pi \varepsilon_{0}\varepsilon {*}^{\prime }L}{ln\left(\frac{R_{2}}{R_{1}}\right)},\;\mathrm{for}\;t>0$$

From Equation (18), we can calculate the apparent relative dielectric permittivity, $\varepsilon^{*}{}^{\prime }$, by using the values $\varepsilon_{0}=8.854\mathrm{pF}/m$, $L=18\times 10^{-3}\;m,$ $R_{2}=9\times 10^{-3}\;m$ and $R_{1}=4\times 10^{-3}\;m$, obtaining the following.

(19) $$\varepsilon^{*}{}^{\prime }=0.810\cdot C_{s}\left(pF\right)$$

**Figure 15:**
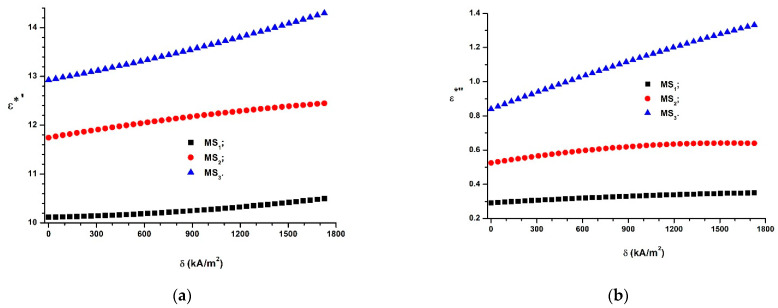
(**a**) Apparent relative dielectric permittivity, $\varepsilon^{*}{}^{\prime }$, as a function of the magnetic field intensity gradient, δ; (**b**) apparent dielectric loss coefficient, $\varepsilon^{*}{}^{\prime \prime }$, as a function of the magnetic field intensity gradient, δ.

If we introduce the functions $C_{s}=C_{s}{\left(\delta \right)}_{CC_{i}}$ (*i* = 1, 2, and 3) from Figure 8a into Equation (19), we obtain the dependences $\varepsilon^{*}{}^{\prime }=\varepsilon^{*}{}^{\prime }{\left(\delta \right)}_{MS_{i}}$ (*i* = 1, 2, and 3), as shown in Figure 15a.

As shown in Figure 15a, the apparent relative dielectric permittivity depends on the volumetric fraction of CIPs and increases when the magnetic field intensity gradient increases.

The functions $\varepsilon^{*}{}^{\prime }=\varepsilon^{*}{}^{\prime }{\left(\delta \right)}_{MS_{i}}$ (*i* = 1, 2, and 3) have the following equation:

(20) $$\varepsilon^{*}{}^{\prime }=\varepsilon^{\prime }+\alpha_{\varepsilon^{*}{}^{\prime }}\delta +\beta_{\varepsilon^{*}{}^{\prime }}\delta^{2}$$

where $\varepsilon^{\prime },\;\alpha_{\varepsilon^{*}{}^{\prime }}$ and $\beta_{\varepsilon^{*}{}^{\prime }}$ are the fitting parameters, provided in Table 6.

The orientation of the CIPs in the magnetic field MSs shown in Figure 13b also impacts the dielectric loss coefficient. Based on the aforementioned observations, we next introduce the quantity $\varepsilon^{*}{}^{\prime \prime }$ called the coefficient of apparent dielectric losses. The connection between $\varepsilon^{*}{}^{\prime \prime }$ and $\varepsilon^{*}{}^{\prime }$ takes the following form (9):

(21) $$\varepsilon^{*}{}^{\prime \prime }=D_{s}\varepsilon^{*}{}^{\prime },\;\mathrm{for}\;t>0$$

where $D_{s}$ is the tangent of the dielectric loss angle.

If we introduce the functions $D_{s}=D_{s}{\left(\delta \right)}_{CC_{i}}$ (*i* = 1, 2 and 3) from Figure 8b and $\varepsilon^{*}{}^{\prime }=\varepsilon^{*}{}^{\prime }{\left(\delta \right)}_{MS_{i}}$ (*i* = 1, 2, and 3) from Figure 15a into Equation (21), we obtain the dependences $\varepsilon^{*}{}^{\prime }=\varepsilon^{*}{}^{\prime }{\left(\delta \right)}_{MS_{i}}$, as shown in Figure 15b.

Figure 15b shows that the functions $\varepsilon^{*}{}^{\prime }=\varepsilon^{*}{}^{\prime }{\left(\delta \right)}_{MS_{i}}$ (*i* = 1, 2 and 3) can be fitted by the following equation:

(22) $$\varepsilon^{*}{}^{\prime \prime }=\varepsilon^{\prime \prime }+\alpha_{\varepsilon^{*}{}^{\prime \prime }}\delta -\beta_{\varepsilon^{*}{}^{\prime \prime }}\delta^{2}$$

where $\varepsilon^{\prime \prime },\;\alpha_{\varepsilon^{*}{}^{\prime \prime }}$ and $\beta_{\varepsilon^{*}{}^{\prime \prime }}$ are the fitting parameters provided in Table 7.

Figure 15b shows that the functions $\varepsilon^{*}{}^{\prime \prime }=\varepsilon^{*}{}^{\prime \prime }{\left(\delta \right)}_{MS_{i}}$ (*i* = 1,2 and 3) depend on the values $\Phi_{CIP}$ (from Table 1). Thus, for $MS_{1}$, sponge $\varepsilon^{*}{}^{\prime \prime }$ increases with an increase in the values of the gradient of the intensity of the magnetic field, δ. Instead, for the $MS_{2}$ sponge, the size of $\varepsilon^{*}{}^{\prime \prime }$ increases with the values of δ to a value of $\delta =1570\;\mathrm{kA}/m^{2}$, at which point the maximum is reached: $\varepsilon_{max}^{*{}^{\prime \prime }}=1.14145$. Above this value of δ, $\varepsilon^{*}{}^{\prime \prime }$ decreases and reaches a minimum value of $\varepsilon_{min}^{*{}^{\prime \prime }}=1.3809$ for $\delta =1769\;\mathrm{kA}/m^{2}$. For the $MS_{3}$ sponge (correlated with the function $R_{1}^{*}=R_{1}^{*}{\left(\delta \right)}_{CC_{3}}$ from Figure 14), dependence $\varepsilon^{*}{}^{\prime \prime }=\varepsilon^{*}{}^{\prime \prime }{\left(\delta \right)}_{MS_{3}}$ is ascending, as shown in Figure 15b.

**Figure 16:**
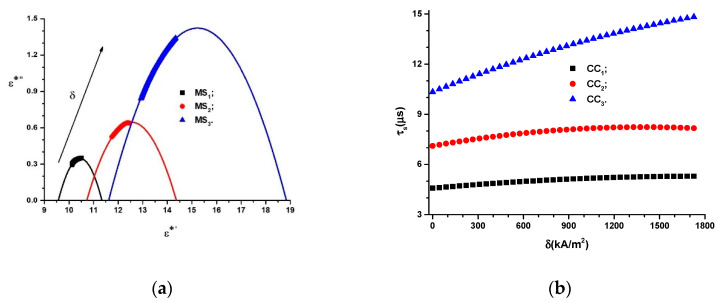
(**a**) Dependence of the apparent relative dielectric permittivity, $\varepsilon^{*}{}^{\prime \prime },$ on the relative dielectric permittivity, $\varepsilon^{*}{}^{\prime }$, of MSs; (**b**) time constant of CCs, $\tau_{s}$, as a function of the magnetic field intensity gradient, δ.

Using the data from Figure 15, we obtained the following function:

(23) $$\varepsilon^{*}{}^{\prime \prime }=\varepsilon^{\prime \prime }+\alpha_{1}\varepsilon^{*}{}^{\prime }-\beta_{1}\varepsilon^{*}{}^{\prime }{}^{2}$$

where the values of the free term,$\;\varepsilon^{\prime \prime }$, and the coefficients $\alpha_{1}$ and $\beta_{1}$ for MSs are the fitting parameters, presented in Table 8. The dependences $\varepsilon^{*}{}^{\prime \prime }=\varepsilon^{*}{}^{\prime \prime }{\left(\varepsilon^{*}{}^{\prime }\right)}_{MS_{s}}$ are depicted in Figure 16a.

The data visualized in Figure 16a allowed us to choose operating points with coordinates $\left(\varepsilon^{*}{}^{\prime },\;\varepsilon^{*}{}^{\prime \prime }\right)$, at which the magnetic field sensors, as well as the sensors and transducers of deformations and mechanical stresses, operate under the preset parameters.

### 3.6. Time Constant of CCs

The time constant for CCs can be calculated using the following equation:

(24) $$\tau \left(\mu s\right)=C_{s}\left(\mathrm{pF}\right)\cdot R_{s}\left(M\Omega \right)$$

By introducing the functions, $C_{s}=C_{s}{\left(\delta \right)}_{C_{i}},\;i=\left(1,2,3\right)$ from Figure 8a into Equation (24), as well as the functions $R_{s}=R_{s}{\left(\delta \right)}_{C_{i}},\;i=\left(1,2,3\right)$ from Figure 10a, we can obtain the dependence $\tau_{s}=\tau_{s}{\left(\delta \right)}_{C_{i}},\;i=\left(1,2,3\right)$, as shown in Figure 16b.

Figure 16b shows that the dependence $\tau_{s}=\tau_{s}{\left(\delta \right)}_{C_{i}},\;i=\left(1,2,3\right)$ has the following form:

(25) $$\tau_{s}=\tau_{s_{0}}+\alpha_{\tau_{s}}\delta -\beta_{\tau_{s}}\delta^{2}$$

where the values of $\tau_{s_{0}},\;\alpha_{\tau_{s}}$ and $\beta_{\tau_{s}}$ for the studied CCs are summarized in Table 9.

Figure 16b shows that the functions $\tau_{s}=\tau_{s}{\left(\delta \right)}_{C_{i}},\;i=\left(1,\;2,\;3\right)$ increase when the amount of the magnetizable phase increases. For the same volumetric fraction of magnetizable microparticles, the time constants, $\tau_{s}$, increase when the magnetic field intensity gradient, δ, increases. Consequently, the storage time of the electricity in CCs can be set raw based on the amount of the magnetizable phase and fine-tuned from the values of the magnetic field strength gradient.

## 4. Conclusions

In this paper, MSs were successfully produced by mechanically doping CIPs into the porous structure of a commercial polyurethane sponge (Figure 3). This small amount of CIPs was well fixed in the cells of the absorbent sponge. When an external magnetic field was applied, the CIPs restructured as aggregates (Figure 12b). Under the action of a magnetic force (Figure 11), the magnetizable metal phase was transported from the sponge volume on the outer surface of the lower cylinder of the CCs. In this manner, with an increase in the value of the average magnetic field intensity and a corresponding increase in the values of the gradient of the magnetic field intensity, the apparent radius of the inner armature (Figure 14) of the CCs increased. The most important resulting effect was modification of the electric resistance (Figure 8a) with the increase in the values of the gradient of the magnetic field intensity and, in particular, in the average magnetic field intensity from the MSs. The ordering of CIPs (Figure 13) in the volume of the porous matrix (PPM) induced magnetodielectric effects in the MSs. The magnetodielectric effects included fine modification of the electric field energy dispersion via the modification of the dielectric permittivity (Figure 15a), the modification of electric field energy absorption (Figure 15b) through modification of the dielectric loss coefficient of the MSs and control of the CC time constant (Figure 16b), which was achieved by adjusting the value of the magnetic field strength gradient. Although the proposed theoretical model is a qualitative one, the obtained results demonstrate that MSs can be used to manufacture low-cost resistive magnetic field sensors. Based on these results, we aim to make magnetizable sponges designed to detect deformations and mechanical stresses for which its sensitivity is controlled by the external magnetic field.

## Figures and Tables

**Table 1:** Weights, volumes and volumetric fractions for $MS_{i}$, where i=1, 2, 3 denote sample numbers.

$MS_{i}$	$m_{PF\;}\left(g\right)$	$m_{CIP\;}\left(g\right)$	$V_{PF\;\;}\left(cm^{3}\right)$	$V_{CIP}\;\left(cm^{3}\right)$	$V_{a}\;\left(cm^{3}\right)$	$\Phi_{PF}\;\left(\%vol.\right)$	$\Phi_{CIP}\;\left(\%vol.\right)$	$\Phi_{a}\;\left(\%vol.\right)$
$MS_{1}$	0.169	0.2525	3.6096	0.0322	4.9678	41.92	0.37	57.71
$MS_{2}$	0.169	0.3380	3.6096	0.0430	4.9570	41.92	0.49	57.59
$MS_{3}$	0.169	0.5070	3.6096	0.0645	4.9355	41.92	0.75	57.33

Note: $m_{PF}\;\mathrm{and}\;m_{CIP}$ are the fiber and CIP weights, respectively; $V_{PF},$ $V_{CIP}$ and $V_{a}$ are the fiber, CIP and air volumes; $\Phi_{PF}\left(\%vol.\right),$ $\Phi_{CIP}\left(\%vol.\right)$ and $\Phi_{a}\left(\%vol.\right)$ are the fiber, CIP and air volumetric fractions.

**Table 2:** The values of $C_{s_{0}},\;\alpha_{C_{s}}$ and $\beta_{C_{s}}$ for the $CC_{s}$ capacitors calculated from the second-order fitting of data from Figure 8a.

$CC_{s}$	$C_{s_{0}}\left(\mathrm{pF}\right)$ $/\upsilon_{C_{s}}$	$\alpha_{C_{s}}\left(\mathrm{pF}\cdot m^{2}/\mathrm{kA}\right)$ $/\upsilon_{C_{s}}$	$\beta_{C_{s}}\left(\left(\mathrm{pF}\cdot m^{4}/kA^{2}\right)\right)$ $/\upsilon_{C_{s}}$
$CC_{1}$	12.49874/0.00644	8.40272$\times 10^{-5}$/$1.52462\times 10^{-5}$	1.09443$\times 10^{-7}$/$7.69947\times 10^{-9}$
$CC_{2}$	14.51244/0.02785	5.27391$\times 10^{-4}$/$6.59306\times 10^{-5}$	1.012271$\times 10^{-7}$/$3.329565\times 10^{-8}$
$CC_{3}$	15971/0.03966	7.39757$\times 10^{-4}$/$9.38924\times 10^{-5}$	1.39335$\times 10^{-7}$/$4.74166\times 10^{-8}$

Here, $\upsilon_{C_{s}}$ is the standard error.

**Table 3:** The values of $R_{s_{0}},\;\alpha_{D_{s}}$ and $\beta_{D_{s}}$ for the $CC_{s}$ capacitors calculated from the second-order fitting of data from Figure 8b.

$CC_{s}$	$D_{s_{0}}$ $/\upsilon_{D}$	$\alpha_{D_{s}}\left(m^{2}/\mathrm{kA}\right)$ $/\upsilon_{D_{s}}$	$\beta_{D_{s}}\left(m^{4}/kA^{2}\right)$ $/\upsilon_{D_{s}}$
$CC_{1}$	0.02881/5.69773$\times 10^{-5}$	5.06593$\times 10^{-6}$/6.07483$\times 10^{-7}$	1.42198$\times 10^{-9}/$6.81121$\times 10^{-11}$
$CC_{2}$	0.04469/2.56596$\times 10^{-4}$	1.018025$\times 10^{-5}$/4.227$\times 10^{-6}$	3.65438$\times 10^{-9}$/3.06785$\times 10^{-10}$
$CC_{3}$	0.06503/4.27432$\times 10^{-4}$	2.29278$\times 10^{-5}$/1.01193$\times 10^{-6}$	3.83616$\times 10^{-9}/5.11035\times 10^{-10}$

Here $\upsilon_{D_{s}}$ is the standard error.

**Table 4:** Comparative values of equivalent electrical capacities of EDs.

EDs	Equivalent Electrical Capacities	Reference
CCs	$C_{s}=12.8\;\mathrm{pF}$ for $MS_{1}$ (Table 1) at $0\leq \delta \left(\mathrm{kA}/m^{2}\right)\leq 1769$ si *f* = 1 kHz $14.5\leq C_{s}\left(\mathrm{pF}\right)\leq 15.5$ for $MS_{2\;}$(Table 1) 1 at $0\leq \delta \left(\mathrm{kA}/m^{2}\right)\leq 1769$ si *f* = 1 kHz $16\leq C_{s}\left(\mathrm{pF}\right)\leq 19.7$ for $MS_{3\;}$(Table 1) 1 at $0\leq \delta \left(\mathrm{kA}/m^{2}\right)\leq 1769$ si *f* = 1 kHz.	Present work Figure 8a
MACs	$70\leq C_{p}\left(\mathrm{pF}\right)\leq 500$ for $MAC_{1\;}$ with $\Phi_{CIP}=17\;vol.\%,\;\Phi_{nBT}=0.0\;vol.\%,\;\Phi_{CF}=83.0\;vol.\%$ at 0≤H(kA/m)≤400 and f=1 kHz $250\leq C_{p}\left(\mathrm{pF}\right)\leq 1500$ for $MAC_{2\;}$ with $\Phi_{CIP}=\Phi_{nBT}=14.6\;vol.\%,\;\Phi_{CF}=70.8\;vol.\%\;$ at 0≤H(kA/m)≤400 and f=1 kHz $250\leq C_{p}\left(\mathrm{pF}\right)\leq 10500$ for $MAC_{3\;}$ with $\Phi_{CIP}=12.7\;vol.\%,\;\Phi_{nBT}=25.4\;vol.\%,\;\Phi_{CF}=61.9\;vol.\%\;$ at 0≤H(kA/m)≤400 and f=1 kHz	[29]
hMCs	$175\leq C_{p}\left(\mathrm{pF}\right)\leq 420$ for $hMC_{1\;}$ with $\Phi_{GB}=17.24\;\;wt.\%,\;\Phi_{CIP}=16.55\;wt.\%,\;\Phi_{SO}=66.21\;wt.\%,\;\Phi_{\mu F}=0.00\;wt.\%,\;\;$ at 0≤H(kA/m)≤320 si *f* = 1 kH and f=1 kHz $120\leq C_{p}\left(\mathrm{pF}\right)\leq 345$ for $hMC_{2\;}$ with $\Phi_{GB}=17.24\;wt.\%,\;\Phi_{CIP}=16.55\;wt.\%,\;\Phi_{SO}=63.73\;wt.\%,\;\Phi_{\mu F}=2.48\;wt.\%,\;\;\;$ at 0≤H(kA/m)≤320 and f=1 kHz $100\leq C_{p}\left(\mathrm{pF}\right)\leq 300$ for $hMC_{3\;}$ with $\Phi_{GB}=17.24\;wt.\%,\;\Phi_{CIP}=16.55\;wt.\%,\;\Phi_{SO}=61.25\;wt.\%,\;\Phi_{\mu F}=4.96\;wt.\%\;$ at 0≤H(kA/m)≤320 and f=1 kHz $80\leq C_{p}\left(\mathrm{pF}\right)\leq 120$ for $hMC_{4\;}$ with $\Phi_{GB}=17.24\;wt.\%,\;\Phi_{CIP}=16.55\;wt.\%,\;\Phi_{SO}=58.77\;wt.\%,\;\Phi_{\mu F}=7.44\;wt.\%,\;\;\;\;$ at 0≤H(kA/m)≤320 and f=1 kHz	[30]

Here: MACs are flat capacitors $\left(30\times 30\times 1.20{\;\mathrm{mm}}^{3}\right)$, with membranes based on CIP, cotton fibers (CT) and barium titanate nanoparticles (nBT); hMCs are flat capacitors $\left(30\times 30\times 0.42{\;\mathrm{mm}}^{3}\right)$, with membranes based on cotton fabrics (GT), silicone oil (SO) and iron microfibers (μF) obtained as described in [31]; $\Phi_{CIP},\Phi_{nBT},\;\Phi_{CF},\;\Phi_{GB}$, $\Phi_{SO}$ ${\mathrm{and}\;\Phi }_{\mu F}$ are the volumetric fractions of CIP, nBT, CF, CB, SO and μF, respectively.

**Table 5:** The values of $\varepsilon^{\prime }$ and $\varepsilon^{\prime \prime }$ for the MSs.

$MS_{i}$	$\varepsilon^{\prime }$	$\varepsilon^{\prime \prime }$
$MS_{1}$	10.1239794	0.291671846514
$MS_{2}$	11.7550764	0.525334364316
$MS_{3}$	12.93651	0.8412612453

**Table 6:** The values of $\varepsilon^{\prime },\;\alpha_{\varepsilon^{*}{}^{\prime }}$ and $\beta_{\varepsilon^{*}{}^{\prime }}$ for MS sponges.

$MS_{i}$	$\varepsilon^{\prime }$ $/\upsilon_{\varepsilon^{*\prime }}$	$\alpha_{\varepsilon^{*\prime }}$ $/\upsilon_{\varepsilon^{*\prime }}$	$\beta_{\varepsilon^{*\prime }}$ $/\upsilon_{\varepsilon^{*\prime }}$
$MS_{1}$	18.01068434/2.74233$\times 10^{-15}$	1.21084$\times 10^{-4}$/7.16058$\times 10^{-18}$	1.57708$\times 10^{-7}$/3.91903$\times 10^{-21}$
$MS_{2}$	20.91242604/1.4432$\times 10^{-16}$	0.001/3.76839$\times 10^{-19}$	1.61782$\times 10^{-7}$/2.06246$\times 10^{-22}$
$MS_{3}$	23.014211/8.56713$\times 10^{-16}$	0.00107/2.23699$\times 10^{-18}$	2.00781$\times 10^{-7}$/1.22432$\times 10^{-21}$

Here, $\upsilon_{\varepsilon^{*}{}^{\prime }}$ is the standard error.

**Table 7:** The values of $\varepsilon^{\prime \prime },\;\alpha_{\varepsilon^{*}{}^{\prime \prime }}$ and $\beta_{\varepsilon^{*}{}^{\prime \prime }}$ for MSs.

$MS_{i}$	$\varepsilon^{\prime \prime }$ $/\upsilon_{\varepsilon^{*\prime \prime }}$	$\alpha_{\varepsilon^{*\prime \prime }}$ $/\upsilon_{\varepsilon^{*\prime \prime }}$	$\beta_{\varepsilon^{*\prime \prime }}$ $/\upsilon_{\varepsilon^{*\prime \prime }}$
$MS_{1}$	0.5188/1.93415$\times 10^{-6}$	9.46878$\times 10^{-5}$/5.05033$\times 10^{-9}$	1.9994$\times 10^{-8}$/2.76408$\times 10^{-12}$
$MS_{2}$	0.93367/3.22217$\times 10^{-6}$	2.64798$\times 10^{-4}$/8.41352$\times 10^{-8}$	8.43646$\times 10^{-8}$/4.60478$\times 10^{-11}$
$MS_{3}$	1.49618/2.22621$\times 10^{-6}$	5.99923$\times 10^{-4}$/5.81293$\times 10^{-9}$	5.35567$\times 10^{-8}$/3.18145$\times 10^{-11}$

Here, $\upsilon_{\varepsilon^{*}{}^{\prime \prime }}$ is the standard error.

**Table 8:** The values of $\varepsilon^{\prime \prime },\;\alpha_{1}$ and $\beta_{1}$ for MSs.

$MS_{i}$	$\varepsilon^{\prime \prime }$ /ν	$\alpha_{1}$ /ν	$\beta_{1}$ /ν
$MS_{1}$	−47.86851/0.98815	9.22566/0.10782	0.44131/0.00524
$MS_{2}$	−29.96986/0.21044	4.88146/0.03473	0.19458/0.00143
$MS_{3}$	−23.85768/0.07655	3.32095/0.01126	0.10906/4.13649$\;\times 10^{-4}$

Here, ν is the standard deviation.

**Table 9:** The values of $\tau_{s_{0}},\;\alpha_{\tau_{s}}$ and $\beta_{\tau_{s}}$ resulting from Figure 16b.

$CC_{s}$	$\tau_{s_{0}}\;\left(\mu s\right)$ $/\upsilon_{C_{s}}$	$\alpha_{C_{s}}\;\left(\mu s/\mathrm{kA}/m^{2}\right)$ $/\upsilon_{\tau_{s}}$	$\beta_{C_{s}}\;\left(\mu s/kA^{2}/m^{4}\right)$ $/\upsilon_{\tau_{s}}$
$CC_{1}$	12.49874/0.00644	8.40272$\times 10^{-5}$/$\;1.52462\times 10^{-5}$	1.09443$\times 10^{-7}$/$\;7.69947\times 10^{-9}$
$CC_{2}$	14.51244/0.02785	5.27391$\times 10^{-4}$/$\;6.59306\times 10^{-5}$	1.012271$\times 10^{-7}$/$\;3.329565\times 10^{-8}$
$CC_{3}$	15.971/0.03966	7.39757$\times 10^{-4}$/$\;9.38924\times 10^{-5}$	1.39335$\times 10^{-7}$/$\;4.74166\times 10^{-8}$

Here, $\upsilon_{\tau_{s}}$ is the standard deviation.

## Data Availability

Not applicable.
